# Cross‐sectional evaluation of early childhood experiences and AD biomarkers in older adults

**DOI:** 10.1002/alz.086628

**Published:** 2025-01-09

**Authors:** Marissa L Streitz, Quoc Bui, Chengjie Xiong, Krista L. Moulder, John C. Morris

**Affiliations:** ^1^ Washington University School of Medicine, St. Louis, MO USA; ^2^ The Charles F. and Joanne Knight Alzheimer Disease Research Center, St. Louis, MO USA; ^3^ Washington University in St. Louis, School of Medicine, St. Louis, MO USA

## Abstract

**Background:**

Calculating an individual’s risk for preclinical and symptomatic Alzheimer disease (AD) involves considering their experiences across the lifespan. This includes assessment of childhood experiences as risk factors for dementing disorders in later life.

**Method:**

The Knight Alzheimer Disease Research Center (ADRC) examined the relationship of well‐established AD biomarkers with childhood experiences as reported by research participants. ANCOVA was used to model biomarker outcomes as a function of childhood events, adjusting for covariates such as age, education, cognitive status, presence of APOE e4, race, sex, and fasting status (for plasma only). Amyloid‐β (Aβ) and tau biomarkers (Aβ42, Aβ40, p‐tau181, p‐tau217) were measured in the cerebrospinal fluid (CSF) and plasma, cerebral amyloid and tau uptakes with positron emission tomography (PET) scans, and MRI scans assessed hippocampal and cortical volumes.

**Result:**

There were 656 participants; 314 were female (56%) and 80 (14%) self‐identified as Black. The mean age was 75.2 ± 8.9 years, and 90% of the participants were cognitively unimpaired (CDR® 0). Those who reported attending a segregated school during childhood demonstrated lower hippocampal volume (p = 0.0005) and cortical thickness (p = 0.0028), as well as higher plasma tau181 (p = 0.037). Participants who attended a private school had lower levels of tauopathy as measured by tau PET compared to those who attended public school (p = 0.036). Frequently experiencing food insecurity throughout early life was related to higher levels of CSF p‐tau181 (p< 0.0001), total tau (p<0.0001), and PET tauopathy (p = 0.03) as well as lower levels of CSF Aβ42/Aβ40 (p = 0.002). Higher levels of CSF total tau (p = 0.0003) and p‐tau181 (p = 0.0015) were found for those who experienced an intensely frightening event as a child. Participants who reported frequent physical punishments showed a trend toward lower hippocampal volumes (p = 0.077). Lower levels of CSF p‐tau181 (p = 0.037) and plasma tau217 (p = 0.029) were found among those who frequently felt special, loved, supported, protected, and close to their families during adolescence. All findings remained significant after adjustment for covariates except for the biomarkers for segregated school attendance.

**Conclusion:**

Early life experiences, both positive and negative, may be associated with the development and presence of AD biomarkers in later life.

